# Burden of chronic kidney disease in Nepal: An analysis of the burden of disease from 1990 to 2019

**DOI:** 10.1371/journal.pgph.0001727

**Published:** 2023-07-19

**Authors:** Achyut Raj Pandey, Anil Poudyal, Bikram Adhikari, Niraj Shrestha

**Affiliations:** 1 Development and Innovation Department, Research, Health Research and Social Development Forum (HERD), Janakpur, Nepal; 2 Research Section, Nepal Health Research Council, Kathmandu, Nepal; 3 Research Section, Social Development and Promotion Center, Kathmandu, Nepal; PLOS: Public Library of Science, UNITED STATES

## Abstract

Chronic kidney disease (CKD) has emerged as one of the major public health concerns. The increasing prevalence of its correlates such as obesity, diabetes, and hypertension has been, due in part responsible for the increased burden. However, very few studies have presented the comprehensive data on burden of disease particularly in developing countries like Nepal. In this study, we have performed an analysis on prevalence, mortality, years lived with disability (YLDs), years of life lost (YLLs) and disability-adjusted life years (DALYs) attributable to CKD in Nepal using Global Burden of Disease (GBD) Study 2019. The GBD 2019 study provides estimation of the prevalence, mortality rates, YLDs, YLLs and DALYs due to 369 different disease and 87 risk factors for 204 countries and territories across the world. In this study, we present Nepal specific data on prevalence, mortality, YLDs, YLLs and DALYs related to CKD. In 2019, there were 1,895,080 prevalent cases of CKD with 5,108 deaths, and a total of 168,900 DALYs were attributable to CKD. Age-standardized prevalence rate of CKD increased from 5,979.1 cases per 100,000 population (95% UI: 5539.7, 6400.4) in 1990 to 7,634.1 cases per 100,000 population (95% UI: 7138.8, 8119.4) in 2019 with higher prevalence in males. Similarly, the age-standardized mortality due to CKD increased for both sexes from 0.8 deaths per 100,000 population (95% UI: 0.6, 1.0) in 1990 to 2.6 deaths per 100,000 population (95% UI: 2.0, 3.3) in 2019. The burden of CKD as a percentage of total DALYs was 0.5% (95% UI: 0.4, 0.6) in 1990 and increased to 1.8% (95% UI: 1.4, 2.2%) in 2019. Kidney dysfunction, high systolic blood pressure, high fasting plasma glucose, high body mass index, low temperature, lead exposure, diet high in sodium, and high temperature were found to be the major risk factors for CKD. The study reveals that Nepal has a high and rising burden of CKD. Innovative strategies for prevention of CKD including health system preparedness for treatment services are required to respond to the rising burden of CKD.

## Introduction

Chronic kidney disease (CKD) is recognized as a public health concern [1]. In general, CKD has been defined as “sustained damage of renal parenchyma that leads to chronic deterioration of renal function and may gradually progress to end-stage renal disease” [2]. However, technically, CKD has been defined as a condition for a minimum of 3 months which is marked by glomerular filtration rate (GFR) below 60 mL/min per 1.73 m2 and/or when ratio of albumin to creatinine in urine is above 30 mg/g irrespective of any underlying cause [3,4].

In 2019, it was estimated that there were 697 million cases (95% UI: 650, 741) of CKD globally [5]. This figure has more than doubled since 1990 when there were an estimated 341 million cases (95% UI: 316, 366). In 2019, about 1.43 million deaths (95% UI: 1.31, 1.52) were estimated to have resulted from CKD. The estimated mortality was 28.8% (95% UI: 22.3, 35.3) higher than 2010 estimates. Meanwhile, between 2010 and 2019, the disability-adjusted life years (DALYs) from CKD rose by 20.1%. The disease was responsible for 41.5 million (95% UI: 38.3, 45.0) DALYs in 2019 [4]. Globally, by 2040, CKD is predicted to be among the top five cause of years of life lost (YLLs) [6].

Studies suggest that, from high to low income countries, hypertension and diabetes mellitus (DM) are two major causes of CKD [4,7]. Other cause include high cholesterol, glomerulonephritis, kidney infections, polycystic kidney disease, blockages in the urinary tract, cardiovascular diseases (CVDs) and use of certain drugs such as non-steroidal anti-inflammatory drugs [7,8]. Few studies on the burden attributable to specific illness and even fewer studies on the burden of CKD are conducted in low and middle-income countries like Nepal [9]. Although few previous studies have reported the prevalence of CKD in Nepal [10,11], the data on mortality attributable to CKD and complete picture of the burden of disease are not available. In this context, we have performed an analysis on prevalence, mortality, YLDs, YLLs and DALYs attributable to CKD in Nepal using Global Burden of Disease (GBD) Study 2019.

## Methods

We obtained data from GBD study 2019, coordinated by the Institute for Health Metrics and Evaluation (IHME) for this analysis. The GBD 2019 is a systematic analysis that aims to estimate the burden of 369 diseases and 87 risk factors across 204 countries and territories. The study provides the estimates for mortality rates, YLDs, YLLs, and DALYs associated with different disease and risk factors. From GBD 2019 study, we have extracted Nepal-specific data for CKD prevalence, YLLs, YLDs, DALYs, and mortality [5].

The GBD study uses data sources such as the census, civil registration and vital statistics, disease registries, household surveys, health service utilization, air pollution monitors, disease notification, among others. The study views CKD as both a metabolic risk factor and a disease. For the purposes of this study, CKD is defined as having an estimated GFR of less than 60 mL/min per 1.73 m^2^ [12].

The 2019 edition of the GDB study attributed each death to a single underlying cause using the international classification of diseases and then classified it into a four-level mutually exclusive and collectively exhaustive GBD cause list. The list includes communicable, maternal, neonatal, and nutritional disorders (CMNN diseases), non-communicable diseases (NCDs), and injuries at level 1, while level 2 consists of 22 clusters of disease and injury aggregates. Meanwhile, level 3 and 4 causes represent more specific diseases and injuries [13]. The GBD 2019 estimates the attributable burden for diseases using a framework established for comparative risk assessment (CRA) [12,14].

In this paper, we have presented both all-age and age-standardized rates for prevalence, mortality and overall burden of CKD. Age-standardization was performed using the GBD world population age standard, and non-weighted means were utilized to create a standard population age structure for all countries and territories. The age-standardization method is discussed in greater detail in a previous article from the GBD study [14]. We have reported 95% uncertainty interval (UI) (95% UI) in the article which was estimated taking 1000 draws from the posterior distribution and the 25^th^ and 975^th^-ordered draw constituted 95% uncertainty distribution [12]. Detail methodology of GBD study has been described elsewhere [12,14].

## Results

Table 1 shows the number of prevalent cases, all-ages and age-standardized prevalence rate of CKD in Nepal in both sex, male and female separately. In 2019, there were 1, 895,080 prevalent cases (95% UI: 1767115, 2022768) which is an increase from 726,897 prevalent cases (95% UI: 669748, 784674) in 1990. The age-standardized prevalence of CKD increased from 5,979.1 cases per 100,000 population (95% UI: 5539.7, 6400.4) in 1990 to 7,634.7 cases per 100,000 population (95% UI: 7138.8, 8119.4) in 2019. For most of the years, males had a higher age-standardized prevalence than females. However, there is quite an overlap between the UI of age-standardized prevalence of CKD among males and females.

**Table 1:** Number of CKD cases and prevalence per 100,000 population.

Year	Both sex			Male			Female		
	Number of prevalent cases	Prevalence rate		Number of prevalent cases	Prevalence rate		Number of prevalent cases	Prevalence rate	
		All ages	Age-standardized		All ages	Age-standardized		All ages	Age-standardized
1990	726897 (669748, 784674)	3720.7 (3428.2, 4016.4)	5979.1 (5539.7, 6400.4)	371595 (342453, 401988)	3794.4 (3496.9, 4104.8)	6064.92 (5611.06, 6535.69)	355302 (326583, 384299)	3646.6 (3351.8, 3944.2)	5880.3 (5432.6, 6304.1)
1995	876150 (810688, 939638)	4033.7 (3732.3, 4325.9)	6452.9 (5994.3, 6899.2)	444809 (412590, 479181)	4088.4 (3792.3, 4404.3)	6517.04 (6029.08, 7019.98)	431342 (398555, 464622)	3978.7 (3676.3, 4285.7)	6379.5 (5910.4, 6809.3)
2000	1026952 (950952, 1102226)	4281 (3964.2, 4594.8)	6630.4 (6162.7, 7072)	518363 (477572, 560046)	4316.2 (3976.5, 4663.2)	6667.75 (6188.24, 7146.56)	508589 (470219, 546488)	4245.8 (3925.4, 4562.1)	6585.2 (6099.1, 7048.4)
2005	1172187 (1091625, 1259224)	4511.3 (4201.2, 4846.3)	6637.5 (6209.1, 7091.9)	580734 (540061, 627814)	4512 (4196, 4877.8)	6619.1 (6181.9, 7089.41)	591453 (548887, 635882)	4510.6 (4186, 4849.4)	6649.6 (6222.8, 7124.1)
2010	1379662 (1288964, 1479370)	4984.9 (4657.2, 5345.2)	6886.6 (6450.1, 7377.8)	671846 (626661, 722627)	4979.7 (4644.8, 5356.1)	6887.87 (6416.64, 7378.45)	707816 (658651, 761233)	4989.9 (4643.3, 5366.5)	6879.6 (6431.5, 7369.1)
2015	1609585 (1502743, 1720003)	5507.1 (5141.6, 5884.9)	7122.9 (6671.2, 7597.1)	764604 (710861, 818884)	5442 (5059.5, 5828.4)	7110.45 (6622.46, 7586.28)	844981 (786641, 905228)	5567.4 (5183, 5964.4)	7126.5 (6653.4, 7601.4)
2019	1895080 (1767115, 2022768)	6230.5 (5809.7, 6650.3)	7634.7 (7138.8, 8119.4)	886509 (827506, 949887)	6114.8 (5707.8, 6551.9)	7635.88 (7109.01, 8160.95)	1008571 (939756, 1077174)	6335.8 (5903.5, 6766.8)	7623.8 (7117.5, 8138.2)

In 2019, among the specific causes, the age-standardized prevalence of CKD due to DM type 2 was 1365.8 cases per 100,000 population (95% UI: 1243.5, 1510.4), CKD due to hypertension was 374.3 cases per 100,000 population (95% UI: 338.0, 416.1), CKD due to glomerulonephritis was 221.3 cases per 100,000 population (95% UI: 176.4, 273.0), and CKD due to DM type 1 was 46.9 cases per 100,000 population (95% UI: 31.2, 73.6). Similarly, the highest proportion of CKD was CKD due to other and unspecified causes with age-standardized prevalence rate of 5,626.4 cases per 100,000 population (95% UI:5245.9, 6006.2) in 2019 (Table 2).

**Table 2:** Number of cases and prevalence of specific CKD per 100,000 population in 2019.

Specific type of CKD	Both sex			Male			Female		
	Number of cases	Prevalence rate		Number of cases	Prevalence rate		Number of cases	Prevalence rate	
		All-ages	Age-standardized		All-ages	Age-standardized		All-ages	Age-standardized
CKD due to DM type 2	332580 (299485, 371910)	1093.4 (984.6, 1222.7)	1365.8 (1243.5, 1510.4)	172179 (153415, 192705)	1187.6 (1058.2, 1329.2)	1508.5 (1357.8, 1675.4)	160401 (143239, 179507)	1007.6 (899.8, 1127.7)	1240 1120.5, 1370.7)
CKD due to Hypertension	81465 (72617, 91908)	267.8 (238.7, 302.2)	374.3 (338.0, 416.1)	42535 (37511, 48559)	293.4 (258.7, 334.9)	415.4 (368, 468.9)	38930 (34146, 44604)	244.6 (214.5, 280.2)	337.4 (300.1, 380.6)
CKD due to glomerulonephritis	66039 (51404, 83620)	217.1 (169, 274.9)	221.3 (176.4, 273.0)	39792 (30280, 50980)	274.5 (208.9, 351.6)	284.3 (222.7, 357.1)	26247 (19339, 34840)	164.9 (121.5, 218.9)	164.5 (124.7, 214.5)
CKD due to DM type 1	14647 (9227, 24283)	48.2 (30.3, 79.8)	46.9 (31.2, 73.6)	7315 (4038, 13011)	50.5 (27.9, 89.7)	48.9 (28.8, 82.3)	7333 (4460, 12308)	46.1 (28, 77.3)	44.1 (27.7, 71.7)
CKD due to other and unspecified causes	1400349 (1305637, 1495458)	4603.9 (4292.5, 4916.6)	5626.4 (5245.9, 6006.2)	172179 (153415, 192705)	4308.8 (3999.3, 4637.5)	5378.7 (4989.6, 5777.1)	775660 (722258, 831291)	4872.7 (4537.2, 5222.1)	5837.8 (5450.9, 6228.5)

(Note: In brackets besides each figures are the lower and upper mark of 95% UI).

There were a total of 5,108 deaths (95% UI: 3581, 6711) from CKD in 2019, which is an increase from 1,728 deaths (95% UI: 1271, 2254) in 1990. From 1990 to 2019, CKD deaths among both male and female in Nepal associated has notably increased. Age-standardized mortality due to CKD increased for both sexes (male and female combined) from 0.8 deaths per 100,000 population (95% UI: 0.6, 1.0) in 1990 to 2.6 deaths per 100,000 population (95% UI: 2.0, 3.3) in 2019. In 2019, males had higher age-standardized mortality than females. (Table 3).

**Table 3:** Number of deaths, CKD Mortality rate per 100,000 and proportion of deaths attributable to CKD.

Year	Both Sex				Male					Female		
	Number of deaths	Mortality rate		% of Deaths	Number of deaths	Mortality rate		% of deaths	Number of deaths	Mortality rate		% of Deaths
		All-ages	Age- standardized			All-ages	Age- standardized			All-ages	Age-standardized	
1990	1728 (1271, 2254)	8.8 (6.5, 11.5)	18.2 (13.3, 24.5)	0.8 (0.6, 1.0)	861 (621, 1250)	8.8 (6.3, 12.8)	19.4 (13.8, 29.5)	0.7 (0.6, 1)	867 (562, 1147)	8.9 (5.8, 11.8)	17.2 (10.9, 23.1)	0.8 (0.5, 1.1)
1995	1910 (1525, 2451)	8.8 (7, 11.3)	18 (14.3, 23.8)	0.9 (0.8, 1.2)	964 (717, 1390)	8.9 (6.6, 12.8)	19.4 ( 14.3, 28.2)	0.9 (0.7, 1.2)	946 (726, 1182)	8.7 (6.7, 10.9)	16.9 (12.8, 21.8)	1 (0.8, 1.2)
2000	2100 (1729, 2662)	8.8 (7.2, 11.1)	17.6 (14.5, 22.7)	1.2 (1.0, 1.5)	1110 (833, 1596)	9.2 (6.9, 13.3)	19.7 (14.9, 28.4)	1.1 (0.9, 1.6)	990 (798, 1214)	8.3 (6.7, 10.1)	15.9 (12.7, 19.8)	1.2 (1, 1.4)
2005	2466 (1967, 3156)	9.5 (7.6, 12.1)	17.9 (14.5, 22.9)	1.5 (1.2, 1.8)	1308 (938, 1951)	10.2 (7.3, 15.2)	20 (14.4, 29.4)	1.4 (1.1, 2.1)	1158 (932, 1404)	8.8 (7.1, 10.7)	16.1 (13.1, 19.6)	1.5 (1.2, 1.8)
2010	3200 (2519, 3976)	11.6 (9.1, 14.4)	19.9 (16, 24.5)	1.9 (1.5, 2.2)	1644 (1212, 2337)	12.2 (9, 17.3)	21.7 (16.3, 30.5)	1.7 (1.3, 2.4)	1557 (1126, 1922)	11 (7.9, 13.6)	18.4 (13.3, 22.6)	2 (1.5, 2.4)
2015	4420 (3228, 5752)	15.1 (11, 19.7)	24 (17.6, 31)	2.3 (1.8, 2.9)	2172 (1533, 3334)	15.5 (10.9, 23.7)	25.3 (17.9, 38.6)	2 (1.5, 3.1)	2248 (1424, 2957)	14.8 (9.4, 19.5)	22.9 (14.6, 29.9)	2.6 (1.7, 3.3)
2019	5108 (3581, 6711)	16.8 (11.8, 22.1)	25.1 (17.7, 32.8)	2.6 (2.0, 3.3)	2447 (1674, 3810)	16.9 (11.5, 26.3)	26.1 (18.1, 40.8)	2.3 (1.8, 3.5)	2661 (1626, 3532)	16.7 (10.2, 22.2)	24.2 (14.8, 31.9)	3 (2, 3.8)

(Note: In brackets besides each figures are the lower and upper mark of 95% UI).

### Mortality attributed to CKD

In 2019, CKD due to DM type 2 and CKD due to other and unspecified causes each attributed approximately 0.7% of total deaths. Among other specific CKDs, 0.6% (95% UI: 0.4, 0.8) of total deaths were from CKD due to hypertension, 0.4% (95% UI: 0.3, 0.6) of total deaths were from CKD due to glomerulonephritis, and 0.2% (95% UI: 0.1, 0.3) of total deaths were from CKD due to DM type 1 (Table 4).

**Table 4:** Mortality from specific CKD conditions.

Specific type of CKD	Both sex				Male				Female			
	Number of deaths	Mortality rate per 100,000 population		% of total deaths	Number of deaths	Mortality rate per 100,000 population		% of total deaths	Number of deaths	Mortality rate per 100,000 population		% of total deaths
		All-ages	Age-standardized			All-ages	Age -standardized			All-ages	Age- standardized	
CKD due to DM type 2	1403 (904, 1991)	4.6 (3, 6.5)	7 (4.7, 9.9)	0.7 (0.5, 1)	693 (421, 1127)	4.8 (2.9, 7.8)	7.3 (4.5, 11.7)	0.7 (0.4, 1)	710 (402, 1007)	4.5 (2.5, 6.3)	6.8 (3.9, 9.5)	0.8 (0.5, 1.1)
CKD due to Hypertension	1203 (820, 1661)	4 (2.7, 5.5)	6.5 (4.4, 8.9)	0.6 (0.4, 0.8)	604 (389, 960)	4.2 (2.7, 6.6)	7.1 (4.7, 11.5)	0.6 (0.4, 0.9)	598 (342, 862)	3.8 (2.1, 5.4)	6 (3.5, 8.5)	0.7 (0.4, 0.9)
CKD due to glomerulonephritis	807 (515, 1162)	2.7 (1.7, 3.8)	3.7 (2.4, 5.4)	0.4 (0.3, 0.6)	357 (214, 585)	2.5 (1.5, 4)	3.7 (2.2, 6.2)	0.3 (0.2, 0.6)	450 (255, 661)	2.8 (1.6, 4.2)	3.8 (2.1, 5.7)	0.5 (0.3, 0.7)
CKD due to DM type 1	330 (185, 556)	1.1 (0.6, 1.8)	1.3 (0.7, 2.2)	0.2 (0.1, 0.3)	154 (79, 285)	1.1 (0.5, 2)	1.3 (0.7, 2.5)	0.1 (0.1, 0.3)	176 (88, 291)	1.1 (0.6, 1.8)	1.3 (0.7, 2.2)	0.2 (0.1, 0.3)
CKD due to other and unspecified causes	1366 (874, 1960)	4.5 (2.9, 6.4)	6.5 (4.2, 9.5)	0.7 (0.5, 1)	639 (401, 1052)	4.4 (2.8, 7.3)	6.7 (4.2, 11)	0.6 (0.4, 1)	727 (412, 1057)	4.6 (2.6, 6.6)	6.3 (3.5, 9.1)	0.8 (0.5, 1.2)

(Note: In brackets besides each figures are the lower and upper mark of 95% UI).

The burden of CKD as a percentage of total DALYs was 0.5% (95% UI: 0.4, 0.6) in 1990 and increased to 1.8% (95% UI: 1.4, 2.2%) in 2019. The age-standardized DALYs due to CKD were stable from 1990 to 2010 and then increased from 2015 to 2019 for both males and females (Table 5).

**Table 5:** DALYs due to CKD per 100,000 and proportion of DALYs attributable to CKD.

	Both Sex			Male			Female		
	All-ages	Age- standardized	% of DALYs	All-ages	Age- standardized	% of DALYs	All-ages	Age– Standardized	% of DALYs
1990	360.7 (276.6, 450.5)	557.2 (432.1, 700.3)	0.5 (0.4, 0.6)	336.4 (263, 466.7)	542.1 (412.1, 754.2)	0.4 (0.3, 0.6)	385.2 (267.1, 499.4)	574.3 (401.3, 739.8)	0.5 (0.3, 0.6)
1995	356.1 (295.1, 434.8)	555.9 (457.5, 687.8)	0.6 (0.5, 0.7)	339.4 (267.8, 454.3)	548.3 (426.9, 745)	0.5 (0.4, 0.7)	373 (296.9, 453.6)	565.1 (451.2, 694.8)	0.6 (0.5, 0.7)
2000	342.8 (285.5, 414.2)	532.4 (444.9, 650.3)	0.7 (0.6, 0.8)	343.1 (268.6, 461.4)	547.6 (427.7, 742.6)	0.7 (0.5, 0.9)	342.5 (283.7, 406.7)	518.7 (432.3, 616.8)	0.7 (0.6, 0.8)
2005	349 (284.7, 431.7)	522.3 (426.4, 648.8)	0.9 (0.7, 1.1)	355.1 (266.2, 489.1)	542.1 (404.4, 756.4)	0.8 (0.6, 1.1)	343.1 (279.6, 404)	503.8 (416.5, 591.4)	0.9 (0.8, 1)
2010	406.4 (323.9, 494.7)	571.2 (460.9, 693.9)	1.2 (1, 1.4)	410 (311.5, 562.2)	584.9 (449.6, 804.9)	1.1 (0.9, 1.5)	403 (305.8, 493)	558.4 (427.1, 678.6)	1.2 (0.9, 1.5)
2015	500.4 (381.6, 637.6)	.661.9 (506.4, 841)	1.5 (1.2, 1.8)	492.9 (360.4, 719.9)	663.1 (486.2, 970.9)	1.3 (1, 1.9)	507.4 (344.4, 651.1)	660.2 (450.1, 847.2)	1.6 (1.2, 2)
2019	555.3 (414.4, 713.2)	697.5 (522.1, 897.6)	1.8 (1.4, 2.2)	538.6 (394, 789.5)	693.2 (507.6, 1020.2)	1.6 (1.3, 2.3)	570.5 (382.9, 736.8)	699.6 (471.3, 903.7)	2 (1.4, 2.5)

(**Note:** In brackets besides each figure there are the lower and upper mark of 95% UI).

CKD due to DM type 2 was responsible for 0.4% (95% UI: 0.3, 0.5) of total DALYs in both sexes followed by CKD due to hypertension (0.3%, 95% UI: 0.3, 0.5) and glomerulonephritis (0.3%, 95% UI: 0.2, 0.4). Similarly, CKD due to DM type 1 was responsible for 0.1% of total deaths in males, females, and both sexes combined (Table 6).

**Table 6:** DALYs due to specific CKD conditions.

Specific type of CKD	Both sex			Male			Female		
	DALYs per 100,000		% of DALYs	DALYs per 100,000		% of DALYs	DALYs per 100,000		% of DALYs
	All-ages	age -standardized		All-ages	age -standardized		All-ages	age -standardized	
CKD due to DM type 2	121.3 (81.9, 167)	164 (111.4, 224.1)	0.4 (0.3, 0.5)	126.2 (81, 192.5)	170.9 (111.6, 260.9)	0.4 (0.3, 0.6)	116.9 (70.8, 164.3)	157.7 (95.6, 221.2)	0.4 (0.3, 0.6)
CKD due to hypertension	104.4 (75.1, 140.5)	142.4 (103.3, 191.2)	0.3 (0.3, 0.5)	108.5 (74.3, 164.4)	151.6 (106.3, 228)	0.3 (0.2, 0.5)	100.7 (63, 141.3)	134.2 (83.8, 185.9)	0.4 (0.2, 0.5)
CKD due to glomerulonephritis	100.3 (70.3, 139.5)	116.5 (81.8, 163.8)	0.3 (0.2, 0.4)	92.6 (62.6, 140.6)	110.4 (74.6, 169.7)	0.3 (0.2, 0.4)	107.3 (67.9, 152.7)	121.6 (74.7, 173.8)	0.4 (0.2, 0.5)
CKD due to DM type 1	39.3 (23, 65.8)	46.4 (26.8, 78.1)	0.1 (0.1, 0.2)	37.9 (20.7, 68.7)	45.8 (24.8, 83.3)	0.1 (0.1, 0.2)	40.7 (21.3, 66.6)	46.8 (24.5, 77.2)	0.1 (0.1, 0.2)
CKD due to other and unspecified causes	189.9 (136.8, 252.4)	228.2 (163.5, 303.8)	0.6 (0.5, 0.8)	173.4 (124.1, 256.3)	214.5 (152.1, 316.9)	0.5 (0.4, 0.8)	205 (140.2, 274)	239.3 (163.8, 320.4)	0.7 (0.5, 0.9)

(Note: In brackets besides each figures are the lower and upper mark of 95% UI).

**Fig 1:**
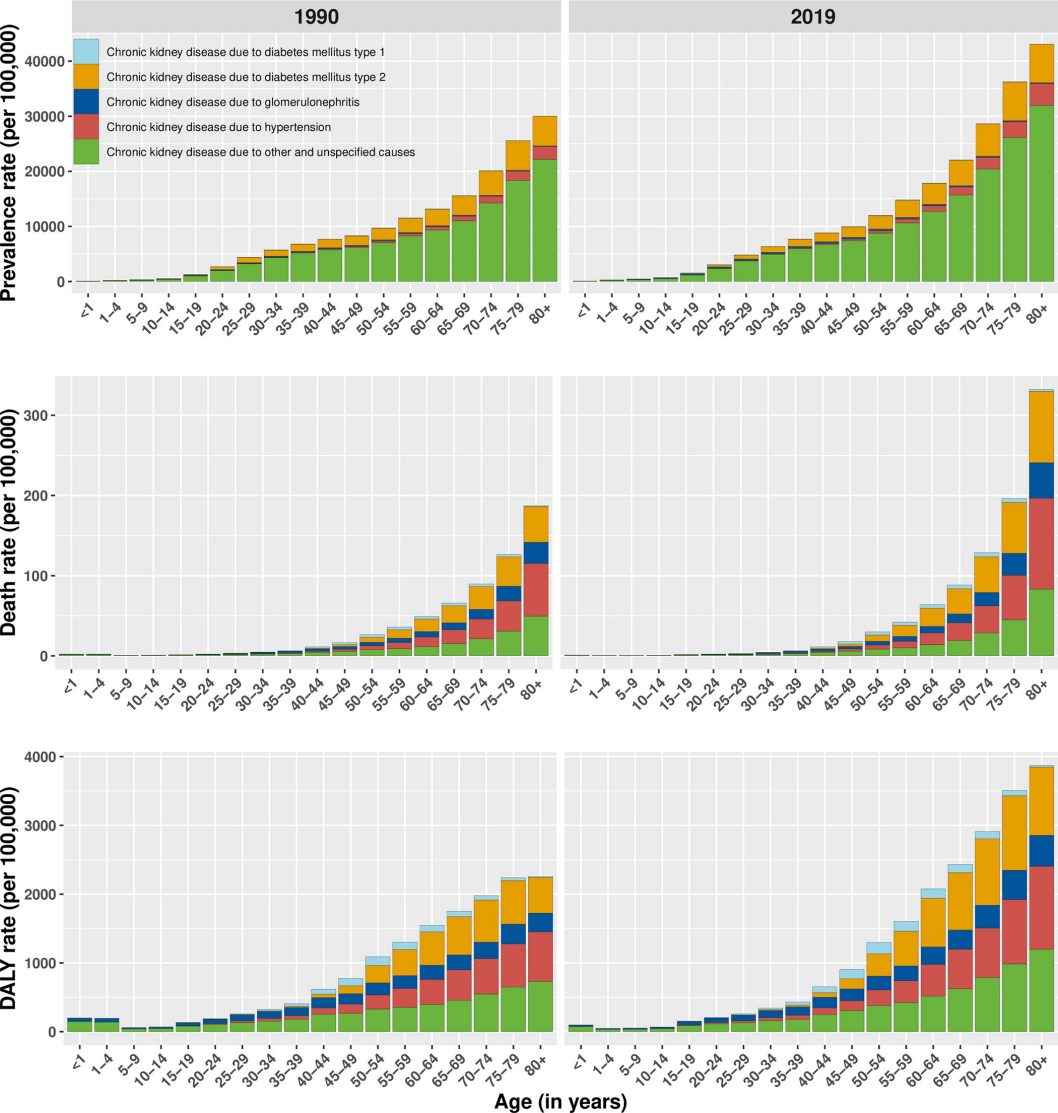
Age-specific prevalence, death rate and DALYs due to CKD in 2019.

The Fig 1 presents the comparison of prevalence rate, death rate, and DALY rate of CKD due to different disease conditions in different age groups between 1990 and 2019. The prevalence rate of CKD due to unspecified cause was highest in age group of 80 years and above with slight rise in the year 2019 compared to 1990. The death rate due to different types of CKDs seem to increase with increasing age. The DALY due CKD due to different diseases has increased in 2019 compared to 1990 for different age group.

**Fig 2:**
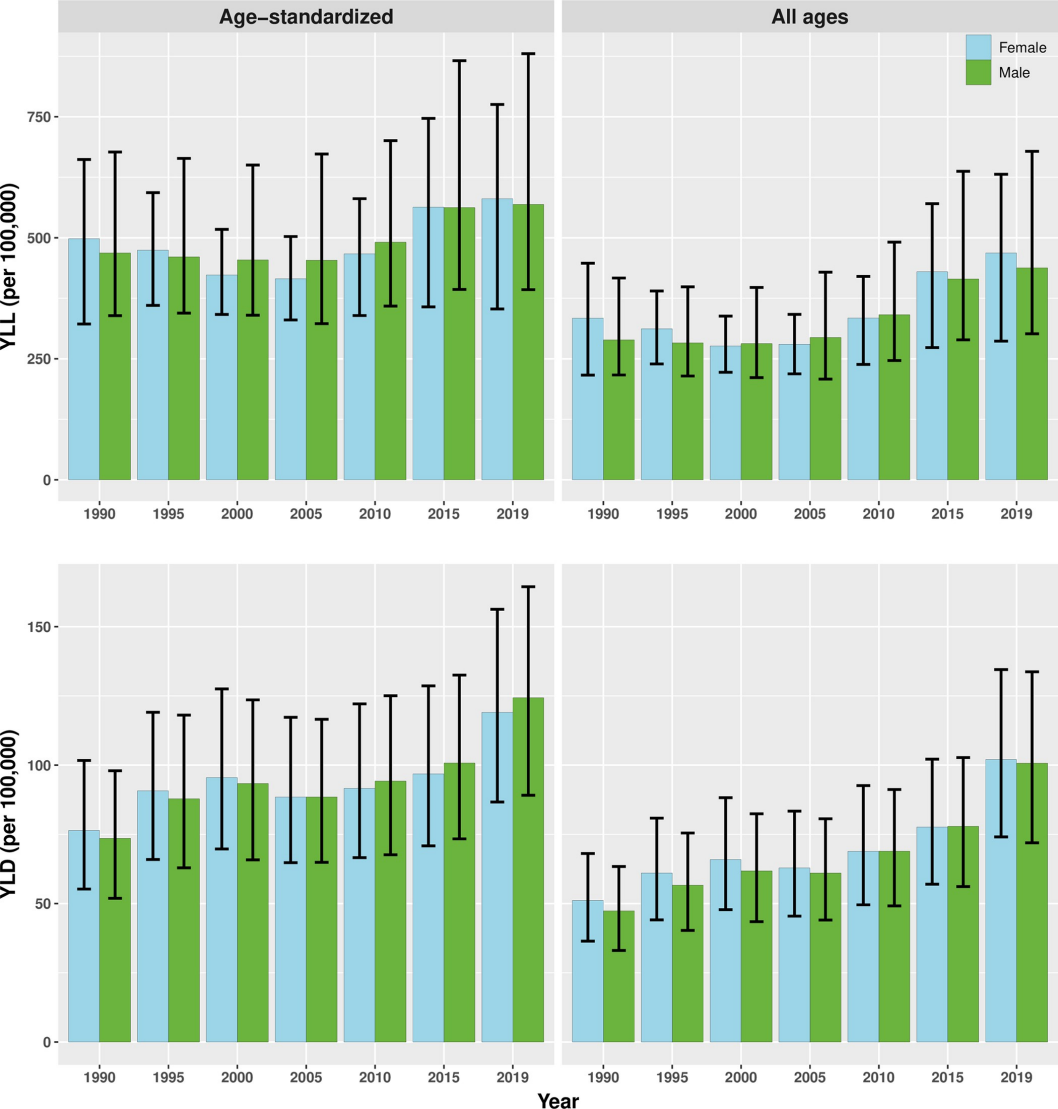
YLDs and YLLs per 100,000 due to CKD from 1990 to 2019.

Fig 2 presents the comparison of age-standardized and all-age YLDs and YLLs in males and females for years from 1990 to 2019. The YLDs and YLLs from CKD seem to have increased for both males and females in the 2019 compared to 1990.

**Fig 3:**
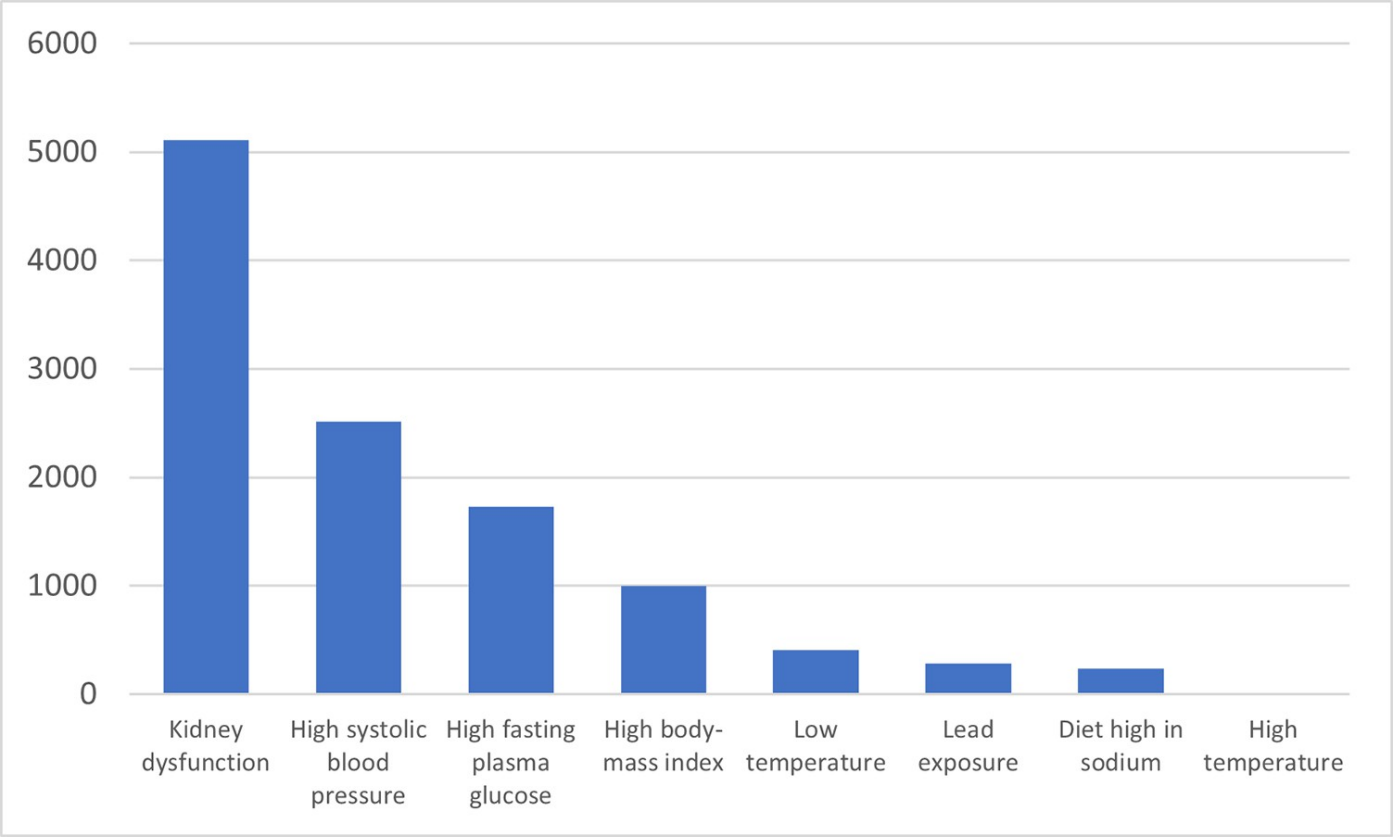
Number of deaths attributable to different risk factors ofCKD in 2019.

Fig 3 shows the major contributing risk factors for CKD. Findings show that kidney dysfunction (5,108 deaths), high systolic blood pressure (2,515 deaths), high fasting plasma glucose (1,733 deaths), high body mass index (1,001 deaths), low temperature (408 deaths) lead exposure (286 deaths), diet high in sodium (238 deaths), and high temperature (4 deaths) were found to be the major risk factors for NCDs.

## Discussion

### Prevalence

Globally, as of 2019, there were an estimated 697 million cases of CKD with the increase in all-age prevalence of CKD by 29·3% since 1990 [5,15]. The rise in prevalence could be linked to rise in prevalence of common risk factors of CKD such as obesity, DM and hypertension [16,17]. In context of Nepal too, similar trends are observable where CKD due to DM type 2 and hypertension are the two leading contributors to all-age and age-standardized prevalence of CKD.

The prevalence of CKD identified in this study (all-age prevalence of 6.26%) is comparable to that indicated in some previous studies where authors have analyzed data from a nationally representative survey (6%) in Nepal [10,18]. In one of the other investigations, Dharan, a city in eastern Nepal, had a prevalence of CKD of 10.6% [11]. In a multi-country study (including Nepal), the prevalence of CKD was reported to be 14.3% in the general population with country specific prevalence of 20.1% in Nepal, 29.9% in China, 16.8% in India, 18.0% in Mongolia, 6.3% in Iran, 23.0% in Nigeria, 25.5% in Moldova, and 5.5% in Bolivia [19]. Previous studies in Nepal have demonstrated wide variation in CKD prevalence [10,11,19]. Similar variations were noted in India, where the prevalence of CKD was reported to be 26% by Anand et al. [20], 13.1% by Huda et al. [21] and 12.8% by Fetema et al. [22]. Within country variations in the prevalence rates could be because of methodological differences and the composition of the population represented in the studies. However, all the studies have demonstrated that a significant proportion of the population bear the burden of CKD, thus indicating the need for efforts in primary prevention of disease, early detection, and treatment of CKD.

### Mortality and DALYs

CKD-related mortality and DALYs have notably increased between 1990 to 2019 in Nepal. They were mostly brought on by a rise in the incidence of risk factors during the period, such as DM type 2 and DM type 1, hypertension, glomerulonephritis, and CKD.

Globally, in 2019, CKD was amongst the top 15 causes of death [5]. Meanwhile, the age-standardized death rate from various types of CKDs increased for CKD due to hypertension by 15.33%, CKD from DM type 2 by 24.62%, CKD from glomerulonephritis by 4.86%, and CKD from DM type 1 by 2.35% [5]. Moreover, in context of Nepal, we observed that the age-standardized deaths have increased by about 38% between 1990 and 2019 with increase in specific types of CKDs. Similarly, in 2019, the age-standardized DALYs for CKD caused by DM type 2 increased by 18.18%, CKD caused by glomerulonephritis increased by 1.59%, and CKD caused by hypertension increased by 10.91% globally [5].

Challenges in accessing appropriate treatment [23], relatively high cost of treatment [24], uncontrolled DM type 2 [25], lack of awareness on CKD causing delayed diagnosis [19,24], lack of health insurance to cover the high cost of treatment [26], uncontrolled hypertension [27] could be the reasons contributing to the rising deaths in low resource setting, such as Nepal. One of the study also indicated that population growth and aging are amongst the other factors leading to burden of CKD [28].

### Prevention of CKD

As per the World Health Organization, lack of early diagnosis and management of NCDs such as DM and hypertension lead to kidney diseases and therefore needs to be addressed as part of a broader response to prevent major NCDs [29]. For instance, CVDs and CKD have common risk factors and therefore, the preventive majors aimed at reducing the burden of CVDs, a major NCD, will work for CKD prevention. Metabolic risk factors were found to have the highest population attributable fraction (PAF) for CKD in 2019, with the top three being elevated systolic blood pressure, increased fasting plasma glucose, and elevated body mass index [12].

While it is indeed value for money, to address CKD prevention efforts as part of broader NCDs prevention efforts in the country, CKD prevention also requires a targeted strategies. The targeted strategy should involve screening high risk groups such as people with DM and hypertension. Although, screening efforts in low-income settings could be challenging financially as well as logistically [30], the cost incurred is far lower than the cost incurred in treating CKD at end stage and overall burden to the health system. Based on studies from multiple countries, Anand et al. suggested that the two-stage screening strategies (annual dipstick screening for proteinuria followed by confirmatory urine protein to creatinine ratio) could be cost-effective [31]. Other initiatives include educating physicians about the increased risk of CKD and the necessity of testing CKD among patients with cardiometabolic disorders to help with the early detection of the condition [31]. However, for screening and early diagnosis program to be effective, they should be accompanied by lifestyle modifications and appropriate treatment services.

People with CKD should be thought of as having a high risk of CVDs, according to prior studies [19,32]. Therefore, more work is required to promote healthy lives particularly aiming to reduce the contributions of high body mass, high fasting plasma glucose, and high systolic blood pressure in CKD. It is undeniably true that therapies to control hypertension and encourage weight loss are linked to lower chances of CKD development and improved outcomes for people already living with CKD. Apart from early detection of CKD and initiating therapies to improve the quality of life among CKD patients, it is critical to design pertinent strategies and focused interventions to prevent and treat hypertension, diabetes, and obesity which seem to have important role in development and progression of the CKD [12].

Some kidney diseases are treatable, depending on the underlying causes. Although there is no known cure for CKD, there are methods to help regulate signs and symptoms, lessen consequences, and delay the illness’s course making early detection of disease an important strategy to improve quality of life [33].

### Policy response and financial support

The study showed a high burden of CKD due to DM type 2 in terms of DALYs and deaths in Nepal. With the rising prevalence of DM and other diseases or risk factors linked to CKD, hypertension and glomerulonephritis, the prevalence of CKD is expected to remain as a challenge in years to come. CKD is associated with substantial morbidity, premature mortality and healthcare costs. Taking cognizance of this issue, Government of Nepal started providing support to CKD patients starting with the financial support in 2011 [34]. For patients with renal impairment, the government provides Nepalese Rupees (NRs). 2,00,000 for a kidney transplant, NRs. 1,00,000 as medicine expenses for post-transplant management and free hemodialysis service is available through the selected hospitals in Nepal [35].

In one of the previous study, the cost of providing financial assistance for an estimated 2900 new patients needing dialysis services was projected to be around US$6.7 million on an annual basis, which was roughly 2.1% of the entire health budget [34]. Without a thorough renal registry, it is difficult to estimate the budget needed to support the program in the future. In addition to money, there are other difficulties in providing all CKD patients with access to healthcare. Nepal currently has 1.4 long-term hemodialysis centers, 0.2 peritoneal dialysis centers, 0.1 transplant centers, and 1.7 nephrologists per million people [36]. However, given the rising demand for care and the dispersed distribution of the population, these numbers may not be adequate in the future. As there are just a few hospitals willing to participate in the government program and give their services, there may be a lengthy waiting period [34]. Patients sometimes have significant non-medical costs as well, such as those related to travel, accommodation and food costs while seeking care among others, because hospitals that provide health services are centralized and primarily located in big cities [37]. Possible increasing burden of CKD prevalence and relatively high cost of treatment reiterate the importance of preventive strategies.

### Strengths and limitations

The study provides the most comprehensive and current estimates of the burden of CKD in Nepal from 1990 to 2019 using prevalence, death, YLDs, YLLs and DALYs. Our study did, however, have certain shortcomings. This study is based on GBD, therefore, all of the basic drawbacks of GBD methodology also apply particularly relating to low availability of high-quality data from the country setting. Despite significant heterogeneity in the risk factors and prevalence of these illnesses, subnational/province-level burden of CKD are not available in this study.

## Conclusions

The study reveals a significant and rising burden of CKD in Nepal. In 2019, among the specific types, CKD due to DM type 2 was found to be most prevalent followed by CKD due to hypertension, CKD due to glomerulonephritis and CKD due to DM type 1. Considering the common risk factors of NCDs and their mutual attribution in the burden, country need to implement broad strategies aiming to prevent overall NCDs as well as interventions specific to CKD. Raising awareness, screening, early diagnosis and treatment services should also be prioritized.

## References

[1] Luyckx VA, Tuttle KR, Garcia-Garcia G, Gharbi MB, Heerspink HJ, Johnson DW, et al. Reducing major risk factors for chronic kidney disease. Kidney international supplements. 2017;7(2):71–87. doi: 10.1016/j.kisu.2017.07.003 30675422 PMC6341126

[2] Akchurin M. Chronic kidney disease and dietary measures to improve outcomes. Pediatric Clinics. 2019;66(1):247–67. doi: 10.1016/j.pcl.2018.09.007 30454747 PMC6623973

[3] Webster AC, Nagler EV, Morton RL, Masson P. Chronic kidney disease. The lancet. 2017;389(10075):1238–52. 10.1016/S0140-6736(16)32064-5 27887750

[4] Institute for Health Metrics and Evaluations. Chronic kidney disease—Level 3 cause [2022 September]. Available from: https://www.healthdata.org/results/gbd_summaries/2019/chronic-kidney-disease-level-3-cause.

[5] Institute for Health Metrics and Evaluations. GBD Compare [2022 September]. Available from: https://vizhub.healthdata.org/gbd-compare/.

[6] Foreman KJ, Marquez N, Dolgert A, Fukutaki K, Fullman N, McGaughey M, et al. Forecasting life expectancy, years of life lost, and all-cause and cause-specific mortality for 250 causes of death: reference and alternative scenarios for 2016–40 for 195 countries and territories. The Lancet. 2018;392(10159):2052–90. doi: 10.1016/S0140-6736(18)31694-5 30340847 PMC6227505

[7] Evans PD, Taal MW. Epidemiology and causes of chronic kidney disease. Medicine. 2011;39(7):402–6.

[8] NHS. Overview: Chronic kidney disease [2022 September]. Available from: https://www.nhs.uk/conditions/kidney-disease/.

[9] Bikbov B, Purcell C, Levey A, Smith M, Abdoli A, Abebe M, et al. GBD Chronic Kidney Disease Collaboration. Global, regional, and national burden of chronic kidney disease, 1990–2017: a systematic analysis for the Global Burden of Disease Study 2017. Lancet. 2020;395(10225):709–33. 32061315 10.1016/S0140-6736(20)30045-3 PMC7049905

[10] Dhimal M, Karki KB, Sharma SK, Aryal KK, Shrestha N, Poudyal A, et al. Prevalence of selected chronic non-communicable diseases in Nepal. Journal of Nepal Health Research Council. 2019;17(3):394–401. doi: 10.33314/jnhrc.v17i3.2327 31735938

[11] Sharma SK, Dhakal S, Thapa L, Ghimire A, Tamrakar R, Chaudhary S, et al. Community-based screening for chronic kidney disease, hypertension and diabetes in Dharan. 2013. 23591297

[12] Ke C, Liang J, Liu M, Liu S, Wang C. Burden of chronic kidney disease and its risk-attributable burden in 137 low-and middle-income countries, 1990–2019: results from the global burden of disease study 2019. BMC nephrology. 2022;23(1):1–12. 34986789 10.1186/s12882-021-02597-3 PMC8727977

[13] Collaborators GA. Global, regional, and national burden of diseases and injuries for adults 70 years and older: systematic analysis for the Global Burden of Disease 2019 Study. bmj. 2022;376. 10.1136/bmj-2021-068208 PMC9316948 35273014

[14] Vos T, Lim SS, Abbafati C, Abbas KM, Abbasi M, Abbasifard M, et al. Global burden of 369 diseases and injuries in 204 countries and territories, 1990–2019: a systematic analysis for the Global Burden of Disease Study 2019. The Lancet. 2020;396(10258):1204–22. doi: 10.1016/S0140-6736(20)30925-9 33069326 PMC7567026

[15] Abraham G, Varughese S, Thandavan T, Iyengar A, Fernando E, Naqvi S, et al. Chronic kidney disease hotspots in developing countries in South Asia. Clinical kidney journal. 2016;9(1):135–41. doi: 10.1093/ckj/sfv109 26798474 PMC4720189

[16] Kovesdy CP. Epidemiology of chronic kidney disease: an update 2022. Kidney International Supplements. 2022;12(1):7–11. doi: 10.1016/j.kisu.2021.11.003 35529086 PMC9073222

[17] Jager KJ, Kovesdy C, Langham R, Rosenberg M, Jha V, Zoccali C. A single number for advocacy and communication—worldwide more than 850 million individuals have kidney diseases. Oxford University Press; 2019. p. 1803–5. 10.1093/ndt/gfz174 31566230

[18] Poudyal A, Karki KB, Shrestha N, Aryal KK, Mahato NK, Bista B, et al. Prevalence and risk factors associated with chronic kidney disease in Nepal: evidence from a nationally representative population-based cross-sectional study. BMJ open. 2022;12(3):e057509. doi: 10.1136/bmjopen-2021-057509 35314475 PMC8938697

[19] Ene-Iordache B, Perico N, Bikbov B, Carminati S, Remuzzi A, Perna A, et al. Chronic kidney disease and cardiovascular risk in six regions of the world (ISN-KDDC): a cross-sectional study. The Lancet Global Health. 2016;4(5):e307–e19. doi: 10.1016/S2214-109X(16)00071-1 27102194

[20] Anand S, Khanam MA, Saquib J, Saquib N, Ahmed T, Alam DS, et al. High prevalence of chronic kidney disease in a community survey of urban Bangladeshis: a cross-sectional study. Globalization and health. 2014;10(1):1–7. 10.1186/1744-8603-10-9 PMC3944963 24555767

[21] Huda MN, Alam KS. Prevalence of chronic kidney disease and its association with risk factors in disadvantageous population. International journal of nephrology. 2012;2012. doi: 10.1155/2012/267329 22848823 PMC3400350

[22] Fatema K, Abedin Z, Mansur A, Rahman F, Khatun T, Sumi N, et al. Screening for chronic kidney diseases among an adult population. Saudi Journal of Kidney Diseases and Transplantation. 2013;24(3):534. doi: 10.4103/1319-2442.111049 23640626

[23] Vos T, Barber RM, Bell B, Bertozzi-Villa A, Biryukov S, Bolliger I, et al. Global, regional, and national incidence, prevalence, and years lived with disability for 301 acute and chronic diseases and injuries in 188 countries, 1990–2013: a systematic analysis for the Global Burden of Disease Study 2013. The lancet. 2015;386(9995):743–800. doi: 10.1016/S0140-6736(15)60692-4 26063472 PMC4561509

[24] Jha V, Garcia-Garcia G, Iseki K, Li Z, Naicker S, Plattner B, et al. Chronic kidney disease: global dimension and perspectives. The Lancet. 2013;382(9888):260–72. doi: 10.1016/S0140-6736(13)60687-X 23727169

[25] Hossain MP, Goyder EC, Rigby JE, El Nahas M. CKD and poverty: a growing global challenge. American Journal of Kidney Diseases. 2009;53(1):166–74. doi: 10.1053/j.ajkd.2007.10.047 19101400

[26] Lozano R, Naghavi M, Foreman K, Lim S, Shibuya K, Aboyans V, et al. Global and regional mortality from 235 causes of death for 20 age groups in 1990 and 2010: a systematic analysis for the Global Burden of Disease Study 2010. The lancet. 2012;380(9859):2095–128. doi: 10.1016/S0140-6736(12)61728-0 23245604 PMC10790329

[27] Ataklte F, Erqou S, Kaptoge S, Taye B, Echouffo-Tcheugui JB, Kengne AP. Burden of undiagnosed hypertension in sub-saharan Africa: a systematic review and meta-analysis. Hypertension. 2015;65(2):291–8. doi: 10.1161/HYPERTENSIONAHA.114.04394 25385758

[28] Xie Y, Bowe B, Mokdad AH, Xian H, Yan Y, Li T, et al. Analysis of the Global Burden of Disease study highlights the global, regional, and national trends of chronic kidney disease epidemiology from 1990 to 2016. Kidney international. 2018;94(3):567–81. 30078514 10.1016/j.kint.2018.04.011

[29] World Health Organization. ActionAction Plan for the Prevention and Control of Non-Communicable Diseases, 2013–2020 Plan for the Prevention and Control of Non-Communicable Diseases, 2013–2020. Geneva, Switzerland: World Health Organization, 2013.

[30] George C, Mogueo A, Okpechi I, Echouffo-Tcheugui JB, Kengne AP. Chronic kidney disease in low-income to middle-income countries: the case for increased screening. BMJ global health. 2017;2(2):e000256. doi: 10.1136/bmjgh-2016-000256 29081996 PMC5584488

[31] Anand S, Thomas B, Remuzzi G, Riella M, Nahas ME, Naicker S, et al. Kidney Disease. Disease Control Priorities 3: Cardiovascular, Respiratory and Related Disorders 2017.

[32] Duni A, Liakopoulos V, Rapsomanikis K-P, Dounousi E. Chronic kidney disease and disproportionally increased cardiovascular damage: does oxidative stress explain the burden? Oxidative medicine and cellular longevity. 2017;2017. doi: 10.1155/2017/9036450 29333213 PMC5733207

[33] Pan American Health Organization WHO, Regional Office for the Americas. Chronic kidney disease. Available from: https://www.paho.org/en/topics/chronic-kidney-disease.

[34] Mcgee J, Pandey B, Maskey A, Frazer T, Mackinney T. Free dialysis in Nepal: Logistical challenges explored. Hemodialysis International. 2018;22(3):283–9. doi: 10.1111/hdi.12629 29446212

[35] Ministry of Health and Population. Guideline for Annual Work Plan and Budgeting in Health Sector at Local Level 2075.

[36] Divyaveer SS, Ramachandran R, Sahay M, Shah DS, Akhtar F, Bello AK, et al. International Society of Nephrology Global Kidney Health Atlas: structures, organization, and services for the management of kidney failure in South Asia. Kidney International Supplements. 2021;11(2):e97–e105. doi: 10.1016/j.kisu.2021.01.006 33981475 PMC8084730

[37] Karki KB, Maskey J, Giri M, Pandey AR, Makai P, Subedi R, et al. Assessment of Chronic Kidney Disease Support Program of Government of Nepal, 2016. Kathmandu: Nepal Health Research Council. 2017.

